# Radial Forearm Free Flap: A Modified Technique for Procurement as a Sentinel Skin Flap

**DOI:** 10.1097/TXD.0000000000001798

**Published:** 2025-04-25

**Authors:** Floris P.C. Kroezen, Sanne Molenkamp, Marijn A. Huijing, Robert A. Pol, Llewellyn Thomas, Leela Sayed, Paul M.N. Werker, Henk Giele

**Affiliations:** 1 Department of Plastic Surgery, University of Groningen, University Medical Center Groningen, Groningen, the Netherlands.; 2 Division of Transplant Surgery, Department of Surgery, University of Groningen, University Medical Center Groningen, Groningen, the Netherlands.; 3 Department of Plastic and Reconstructive Surgery, John Radcliffe Hospital, Oxford University Hospitals NHS Foundation Trust, Oxford, United Kingdom.

## Abstract

**Background.:**

Organ rejection after solid organ transplantation remains a major challenge. The sentinel skin flap (SSF), a vascularized skin flap procured from the donor and transplanted alongside solid organs, has shown promise for early detection of rejection. The radial forearm free flap (RFFF) has a long history of use in reconstructive surgery and offers distinct advantages as SSF in organ donation procedures (ODPs). Until now, the SSF has been procured using the traditional RFFF way. However, an easier, quicker, and safer way is beneficial for logistical and financial reasons. This study presents a modified RFFF procurement technique for ODPs that is simple, quick, and reproducible and can be executed by surgeons who are not familiar with the original RFFF.

**Methods.:**

The traditional RFFF procurement technique was modified using deceased donor models, leading to the development of an SSF technique tailored for application during ODPs.

**Results.:**

A modified technique was developed and presented, enabling SSF procurement to be completed within 40 min. During the procedure, the donor’s arm was positioned above the head or at a 90-degree angle, allowing the creation of a sterile field without interfering with the anesthesiology or organ procurement team. Donor-site closure was achieved using running sutures, ensuring an optimal cosmetic outcome.

**Conclusions.:**

We present a modified RFFF procurement technique, designed to obtain an SSF during ODPs, that we believe can also be executed by surgeons not familiar with this flap, ensuring that the procedure does not interfere with or impede the organ procurement procedure.

Solid organ transplantation (SOT) stands as the primary treatment for individuals with end-stage organ failure. Despite significant advancements since the first kidney transplantation in 1954, rejection remains a major challenge for most organs. Detecting rejection early is critical to prevent chronic transplant dysfunction and organ loss.^1-3^ However, symptoms of rejection are often vague, making biopsies often necessary for diagnosis. Such biopsies are invasive, costly, and carry risks.^4-10^ Moreover, diagnosing rejection through biopsies is complicated by variability between pathologists and difficulties in distinguishing rejection from infection.^11,12^ Possibly, this variability is not solved with the introduction of the SSF. However, additional information provided by the SSF, including its ease of visual assessment, may assist in reaching a diagnosis of rejection earlier. Noninvasive biomarkers offer promising alternatives, but identifying reliable markers remains challenging due to the complex and multifactorial nature of rejection.^13^ These limitations underscore the urgent need for additional diagnostic strategies.

The sentinel skin flap (SSF)—a vascularized skin flap transplanted alongside an organ—offers a promising solution as an innovative tool for early SOT rejection detection. This concept originates from the insights from vascularized composite tissue allografts (VCA), such as face, hand, and upper limb transplants,^14,15^ as well as abdominal wall transplants performed alongside intestinal and multivisceral organ transplants.^15-19^ Unpublished data from the Oxford group suggest improved outcomes in intestinal transplant survival, rejection rates, and reduced infection misdiagnosis in patients receiving both an intestinal transplant and an abdominal wall graft compared with SOT alone. However, discordant rejection episodes between skin and intestinal grafts have been reported, particularly when the grafts originate from different donors. This issue most likely would not arise with an SSF transplanted from the same donor as the organ, maintaining immunological compatibility. However, it is important to note that the concordance of skin flap rejection with SOT rejection remains to be determined.

These studies suggest that SSF can visibly manifest signs of rejection, often preceding those observed in the transplanted organ or the VCA. The SSF’s increased sensitivity to rejection is attributed to the skin’s high immunogenicity, making it an ideal candidate for real-time, noninvasive monitoring.^20,21^

The SSF enables recipients to monitor their skin for visible changes, such as erythema, which may indicate rejection. This allows patients to seek medical evaluation, including skin biopsies, potentially reducing the need for invasive biopsies of transplanted organs. Furthermore, because the SSF often manifests rejection before affecting the solid organ, timely treatment may help minimize rejection-related damage, hereby possibly decreasing patient morbidity and mortality.

Since the 1980s, the radial forearm free flap (RFFF) has been used as a workhorse in reconstructive surgery.^22^ The RFFF is a vascularized fasciocutaneous flap sourced from the volar side of the forearm, supplied by the radial artery and its concomitant veins. It quickly gained popularity owing to its straightforward procurement, consistent anatomy, long and flexible pedicle, and favorable vessel size, making it a highly versatile option for various reconstructive applications.

In the context of organ donation and transplantation, the RFFF offers additional advantages. The donor’s arms can be positioned above the head or at a 90-degree angle, allowing simultaneous procurement of the RFFF and solid organs without procedural interference. Furthermore, the ability to procure bilateral RFFFs and/or the division of a single long flap into 2 SSFs (due to the minimal pedicle length required for transplantation) enhances its utility, potentially obtaining an SSF for multiple organs.

However, the unique demands of organ donation procedures (ODPs) require a tailored approach to RFFF procurement. Unlike elective reconstructive surgeries, which are carefully planned and tailored to the needs of individual patients, ODPs are more urgent, prioritizing solid organ retrieval and achieving primary wound closure with concealable scars. Since the donor is deceased, considerations such as preserving nerves, muscles, and tendons in the donor area are less critical, and the focus can lie solely on the preservation of the vascular pedicle. Furthermore, minimizing warm ischemia time is critical to prevent hypoxic damage and ensure the viability of RFFF, just like solid organ procurement.

In the Netherlands and the United Kingdom, ODPs are typically performed by specialized teams from academic hospitals that travel to regional hospitals. These procedures can span the entire day, including travel. Although plastic surgeons are pivotal in elective SSF procurement, they are generally not involved in ODPs, highlighting the need for a procurement technique that non–plastic surgeons can perform, which is quick and efficient and with minimal risk of damage to the vascular pedicle. This article outlines the steps leading to this modified approach and the actual procedure.

## PATIENTS AND METHODS

### Participants

The idea for SSF was conceived in the United Kingdom, and the first SSF was raised in June 2013 by plastic surgeons and applied during small bowel transplantation in Oxford. The SSF procurement adhered to the traditional RFFF technique.

The Groningen group, which consists of plastic and organ procurement surgeons, aimed to develop a simplified technique for specific use in ODPs. Therefore, 4 deceased human donors, donated for scientific research, were used to practice SSF procurement practices at the Skills Center of the University Medical Center Groningen (UMCG).

To validate the modified technique, it was decided to apply it during ODPs at the UMCG. Ethical approval for SSF procurement during ODPs was granted by the Medical Ethics Review Committee of the UMCG (METc, 2023/526), which classified the study as non-WMO (not subject to the Medical Research Involving Human Subjects Act).

Throughout the development process, all steps strictly adhered to standard donor care and organ procurement protocols. Additionally, during the procurement from donors, explicit written consent from donors’ relatives was obtained by the organ donation coordinator before the ODPs.

### Equipment

SSF procurement was performed using binocular loupe magnification (×2.5–2.8). During the practice sessions at the Skills Center, as well as during the ODPs, standard plastic surgical instruments were used. During the ODPs bipolar diathermy and an automatic clip applier, the Ethicon LigaClip MCA, Small Titanium, 20 clips (ETHICON, Somerville, NJ) were used. The skin was closed using a Monocryl 3-0 (ETHICON, Somerville, NJ) and the linear scar was covered with an adhesive bandage. The SSF was flushed using a 16- or 18-gauge catheter and with the University of Wisconsin cold storage solution (Bridge to Life Ltd, Northbrook, IL). Three sterile bags were used for storage.

## RESULTS

### Development of the Procurement Technique

The initial development and refinement of the technique were done using deceased donor models, allowing practice sessions and adjustments before transitioning to ODPs. The deceased donor sessions showed that incorporating the brachioradialis (BR) tendon into the flap not only provided additional protection for the vascular pedicle but also simplified and accelerated the procurement process.

Subsequently, during the first ODP, it was discovered that including the flexor carpi radialis (FCR) tendon further enhanced the procedure’s efficiency and smoothness, contributing to the overall optimization of the technique. Including these structures in the SSF also facilitates the primary closure of the wound.

### Donor Position and Flap Design

**FIGURE 1.**
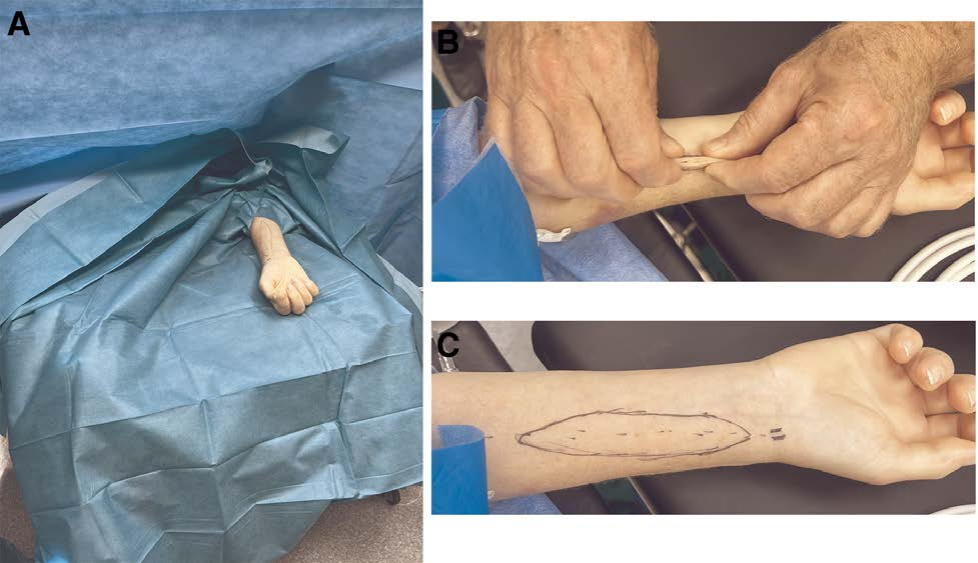
Positioning and planning of the flap. A, Positioning of the donor with the designated arm extended above the head and the distinct surgical fields created with sterile drapes. B, Determining the maximal width of the SSF with a radioulnar skin pinch. C, Planning of the SSF with the course of the radial artery as the axis of the flap. SSF, sentinel skin flap.

The donor is placed in a supine position on the operating table, with the upper extremities elevated above the head or at a 90-degree angle. This positioning facilitates simultaneous access for the plastic surgery and organ procurement teams, as well as the anesthesiology team. Sterile surgical drapes are used to separate the head area from the thorax, creating distinct operative fields (Figure 1A).

For donation after brain death, the radial artery is palpated and marked with a surgical marker at the wrist, and the brachial artery is marked in the cubital fossa. The line connecting these points represents the course of the radial artery and defines the flap axis. Along this line, the skin of the forearm is pinched in an ulnar-to-radial direction to determine the maximum flap width that would allow for primary closure. These margins are marked accordingly, typically resulting in a flap width of 3–4 cm. The flap is then outlined in an elliptical shape, measuring approximately 10–13 cm in length (Figure 1B and C).

During donation after circulatory death, the radial artery is not palpable. Instead, its expected location is identified and marked just radial to the FCR. Similarly, the brachial artery is marked halfway through the cubital fossa, and the line between this point and the expected location of the radial artery at the wrist defines the flap axis. Subsequently, the same steps for determining the maximum flap width and outlining the flap are performed as in donation after brain death cases.

### SSF Procurement During ODP

SSF procurement begins with a longitudinal incision along the ulnar side, extending through the skin and the underlying fat and antebrachial fascia. Bipolar forceps or clips are used to coagulate crossing subdermal and cutaneous vessels. Sutures are used to fix the skin to the fascia to prevent shear that could displace the skin relative to the fascia during the procedure.

**FIGURE 2.**
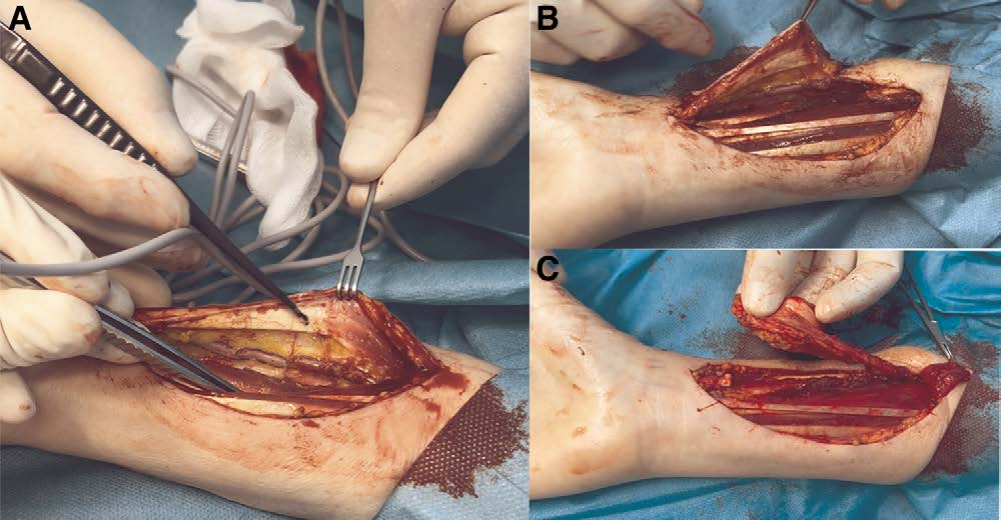
Dissection of the SSF. A, Dissection from the ulnar-to-radial side, indicating the FCR tendon with the forceps. B, Division of the FCR tendon proximally, with the distal part of the tendon included in the SSF. C, Elevation of the flap from the PQ and FPL. The SSF is proximally attached via the vascular pedicle. FCR, flexor carpi radialis; FPL, flexor pollicis longus; PQ, pronator quadratus; SSF, sentinel skin flap.

The dissection proceeds subfascially toward the radial side until the tendon of the FCR is identified (Figure 2A). If the palmaris longus (PL) tendon is present, it is divided distally and retraced proximally. The FCR tendon is divided distally and included in the flap to protect the vascular pedicle (Figure 2B).

Subsequently, the radial artery and its concomitant veins are lifted from the underlying flexor pollicis longus (FPL) and pronator quadratus (PQ) muscles together with the deep fascia. Side branches of the radial artery, which supply the FPL and PQ muscles, are ligated using an automatic clip applier. The FCR tendon is sharply divided proximally to ensure that no muscle tissue is included in the flap.

Next, the skin, subcutis, and fascia of the radial border of the flap are incised while crossing subdermal and cutaneous vessels are coagulated or clipped and divided. The dissection continues subfascially until the tendon of the BR is identified. The BR tendon is divided distally, after which the radial artery and its concomitant veins are clipped on both the donor and flap sides. The BR and vascular pedicle are thereafter carefully elevated from their bed, with meticulous coagulation or clipping of the radial artery side branches. Dissection proceeds from distal to proximal, lifting the flap from the PL and FPL bed until the BR muscle becomes visible, at which point the BR tendon is divided (Figure 2C).

**FIGURE 3.**
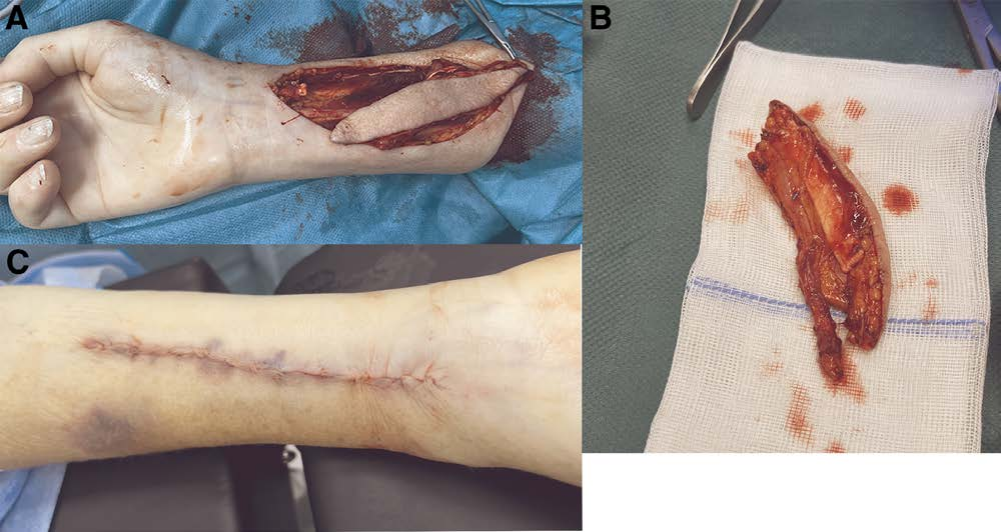
Procurement of the SSF and primary closing of the defect. A, SSF solely attached by its vascular pedicle. B, SSF on the back table. C, Primary closure of the defect. SSF, sentinel skin flap.

At this stage, the flap only remains attached proximally to its vascular pedicle. An extra pedicle length of at least 3 cm is isolated proximal to the skin paddle, with the radial artery separated from its concomitant veins for at least 2 cm (Figure 3A). Clips are applied to the artery and veins on the donor site and sharply divided distal to the clips, completing the RFFF procurement.

The flap is then transferred to a back table (Figure 3B). The defect in the donor forearm is closed in 2 layers using a Monocryl 3-0 suture (Figure 3C), and the wound is covered with a large adhesive bandage.

On the back table, the radial artery is cannulated with a 16- or 18-gauge catheter, which is secured with a ligature. The flap is flushed with 200 mL of University of Wisconsin cold storage solution under 100 cm H_2_O pressure. Finally, the SSF is placed on static cold storage following EuroTransplant and National Health Service Blood and Transplant guidelines, packaged in 3 sterile bags.

## DISCUSSION

Abdominal wall transplantation as an adjunct to small bowel transplantation led to the concept of incorporating an SSF as a monitor for the rejection of solid organs. This study presents a modified RFFF procurement technique specifically adapted for ODPs and proved quick, safe, and executable by non–plastic surgeons.

Since 2013, traditional RFFFs have been successfully procured and transplanted as SSFs in the United Kingdom. The development of an SSF for SOT was subsequently pursued in the Netherlands, where the Groningen group, comprising plastic and organ procurement surgeons, redefined the procurement technique to address the unique requirements of ODPs.

The modified technique involves the procurement of a narrow RFFF centered over the radial artery and the incorporation of FCR and BR tendons into the flap to accelerate procurement and make it easily reproducible. The FCR and BR tendons can easily be removed by the plastic surgeon on the back table before transplantation or after revascularization in the recipient, ensuring that their incorporation into the flap does not impede the insetting of the flap.

These adaptations are believed to enable non–plastic surgeons, who are the primary surgeons during ODPs, to independently procure the SSF either before or after the ODP without disrupting workflow. This approach reduces the logistical complexity and associated costs by eliminating the need for plastic surgeons to be part of the on-call team for ODPs. To ensure broader adoption and consistent outcomes, hands-on training for non–plastic surgeons in fresh tissue laboratory settings and the implementation of standardized protocols are essential. However, when an additional surgeon is available to procure the SSF, positioning the donor’s arms above the head or at a 90-degree angle creates a separate sterile field, allowing for simultaneous accessibility and enabling the surgical teams to work concurrently.

Flap procurement using the modified technique offers significant advantages over traditional RFFF procurement during autologous reconstruction. The absence of the need to preserve tendons and nerves in the donor area simplifies the procedure, making it less time-consuming. Furthermore, because primary closure of the donor site, resulting in a linear wound located only on the volar side of the arm, is still achievable, this discrete wound location minimizes visibility, potentially reducing distress for the relatives.

The technique’s potential for bilateral procurement or division into multiple SSFs significantly enhances its use, especially in multiorgan transplant scenarios. This feature optimizes donor resources and supports improved transplantation outcomes across several recipients.

SSFs not used for transplantation could serve as valuable tools for both preservation and immune response studies using extracorporeal machine perfusion setups. With advancements in machine perfusion expanding ischemia times for organs, the limits of SSFs viability during prolonged preservation remain unclear. Studying SSFs under these conditions could help determine the impact of extended preservation on their viability and identify thresholds for the deterioration of their functionality and usability. Additionally, these perfusion setups provide a controlled environment for investigating immune responses, such as cytokine production and cellular infiltration, simulating transplantation scenarios without transplanting the SSF. This dual-purpose approach could offer critical insights into optimizing preservation techniques and understanding immune dynamics in transplantation research.

It is important to note that procuring an SSF precludes the harvest of the same upper limb or hand for transplantation. Upper limb transplantation typically requires the preservation of major nerves, tendons, and the radial artery, all of which would be compromised during SSF procurement.

The posterior tibial artery flap, as described by Suchyta et al,^23^ offers an alternative for SSF procurement. This approach avoids compromising the upper limb, preserving it for potential transplantation while still providing a reliable marker for monitoring and facilitating biopsies in case of suspected rejection in both SOT and VCA.

While the routine use of SSF in SOT in the Netherlands is still premature, the results of the ongoing randomized controlled trial in the United Kingdom, assessing the utility of SSF in lung transplantation (NIHR 130899), could mark a pivotal change. Upon demonstration of its efficacy in rejection monitoring and subsequent treatment, SSF may become the standard of care in selected SOTs. In the meantime, the procured flaps in the Netherlands will be used for preservation studies.

## CONCLUSIONS

The modified SSF procurement technique provides a fast, efficient, and safe method to procure the flap without impeding organ retrieval; its simplicity enables non–plastic surgeons to learn and implement it effectively. As studies in Oxford continue to assess the role of SSFs in monitoring lung transplant rejection, SSF may become a valuable addition to SOT, improving patient outcomes and streamlining procedural logistics.

## ACKNOWLEDGMENTS

The authors extend their gratitude to the employees of the UMCG Skills Lab for their outstanding preparation and for providing access to their facilities. They also acknowledge the invaluable support of the UMCG organ donation coordinators in obtaining consent for the procurement of the SSF. Most importantly, the authors wish to express their deepest appreciation to the relatives of the donors who graciously gave their consent. This research would not have been possible without their generosity and support.
